# Navigating Vaccine Uncertainty: Anxiety and Fourth Dose Hesitancy Among Saudi Adults

**DOI:** 10.7759/cureus.48766

**Published:** 2023-11-13

**Authors:** Majed M Althomali, Anas S Almalki, Saad M Alotaibi, Abdulelah M Alsalman, Saeed M Alqhatani, Gaber M Shehab, Ahmed S Abdel-Moneim

**Affiliations:** 1 College of Medicine, Taif University, Taif, SAU; 2 Department of Biochemistry, College of Medicine, Taif University, Taif, SAU; 3 Department of Microbiology, College of Medicine, Taif University, Taif, SAU

**Keywords:** coronavirus, covid-19, sars-cov-2, vaccine hesitancy, virology

## Abstract

Background

The continuous evolution of new variants of severe acute respiratory syndrome coronavirus 2 (SARS-CoV-2) and early evidence of declining effectiveness of the third dose over time have generated anxiety and hesitancy regarding vaccinations. The current study aimed to assess anxiety levels and the willingness to receive a fourth dose of the SARS-CoV-2 vaccine. Potential factors leading to this reluctance were also assessed.

Methodology

This was a cross-sectional cohort study conducted among the adult Saudi population. A questionnaire including demographic data, questions regarding Generalized Anxiety Disorder (GAD-7) assessment, and questions related to accepting the vaccine and reasons for hesitancy was employed.

Results

Of the 1,924 participants who responded, 1,033 were males, and 891 were females. Among the respondents, a significant level of anxiety toward receiving the fourth dose of the SARS-CoV-2 vaccine was reported in 1,097 cases, representing 57% of the total, with varying degrees of anxiety. Both gender and age were identified as co-factors contributing to this anxiety. A substantial portion of the participants, 1,369 individuals, accounting for 71.2%, exhibited vaccine hesitancy and reluctance to receive the fourth dose.

Conclusions

Our findings underscore the pressing need for targeted interventions to combat vaccine hesitancy and alleviate associated anxieties, particularly among the adult Saudi population. As we persist in confronting the ongoing challenges brought about by the evolving pandemic, it is crucial that we customize our vaccination campaigns and communication strategies to tackle the apprehensions and hesitations of the Saudi population directly.

## Introduction

Severe acute respiratory syndrome coronavirus 2 (SARS-CoV-2), which emerged in late 2019, is responsible for the global coronavirus disease 2019 (COVID-19) pandemic, a highly contagious respiratory illness that has had a profound impact on public health and the world’s socioeconomic landscape. Understanding the genetic and epidemiological characteristics of SARS-CoV-2 is crucial for the effective management and control of COVID-19, which remains a significant public health concern, prompting extensive research and vaccination efforts worldwide. Point mutation and recombination of the SARS-CoV-2 are natural forms of virus evolution; therefore, new variants of concern (VOCs) will continue to emerge. Many VOCs have been reported, including Alpha (B.1.1.7), Beta (B.1.351), Gamma (P.1), Delta (B.1.617.2), and Omicron (B.1.1.529) [1-5]. Antibodies elicited by previous infections or vaccination can generally neutralize SARS-CoV-2 VOCs; however, there may be some reduction in potency, especially for the Beta, Delta, and Omicron VOCs [6-9]. The circulation of multiple VOCs of SARS-CoV-2 and the evidence of incomplete neutralization presented in the current vaccines against different VOCs have driven several countries to adopt a third dose of the COVID-19 vaccine [6-9]. Early evidence supports the efficacy of the third dose in protecting against Delta and Omicron VOCs [10]. On the other hand, the existing vaccination schedule, which includes three doses, results in broad but incomplete immunity against SARS-CoV-2 variants, including Omicron [11]. In addition, the effectiveness of the third dose has been shown to decline over time [12]. A fourth dose could help increase the level of protection, especially for people in high-risk groups. Therefore, several countries have already recommended a fourth vaccine dose for COVID-19 for the elderly and immunocompromised individuals. For example, the U.S. Food and Drug Administration (FDA) has approved a fourth dose for certain individuals, taking into account the known and potential benefits and risks [13]. A fourth dose is effective in reducing rates of SARS-CoV-2 infection, severe COVID-19, short-term risk of COVID-19-related outcomes, and hospitalizations and deaths due to COVID-19 [14].

Saudi Arabia has approved or authorized the following four vaccines: Pfizer-BioNTech (mRNA vaccines), Moderna (mRNA vaccines), Oxford-AstraZeneca (protein subunit vaccine), and Janssen (viral vector vaccine) [15]. Through March 11, 2023, 68,454,048 doses of the SARS-CoV-2 vaccine were administered to Saudi nationals and residents [16]. The European Centre for Disease Prevention and Control (ECDC) advised a fourth dose of mRNA vaccines for adults aged 80 years and older, considering them to be at the highest risk of severe COVID-19, but found it too early to recommend a fourth dose for those below this age [17]. The Saudi Ministry of Health declared the availability of the fourth dose (second booster dose) four months after the first booster dose with an updated vaccine against mutant SARS-CoV-2 strains. Those eligible included all people over 50 years old, patients with renal failure, patients subjected to bone marrow grafts in the past two years and still under immunosuppressive therapy, patients subjected to organ transplantation and still under immunosuppressive therapy, patients with immunosuppressive conditions, and active cancer patients. An updated dose against mutant SARS-CoV-2 strains is also available as an optional choice for people wishing to obtain it, two months or more after the date of receiving the previous dose.

However, the intention of the public to receive a fourth dose remains unknown. Moreover, a fourth dose of an updated SARS-CoV-2 vaccine containing new prevalent VOCs may be very important, as the emergence of new COVID-19 viral variants is always possible. Therefore, the purpose of this study was to investigate the willingness of the public to receive a fourth dose of the COVID-19 vaccine and its associated factors.

## Materials and methods

Study design and subjects

This cross-sectional study was designed to screen the anxiety among the Saudi population in receiving the fourth dose of the COVID-19 vaccine. An online sample size calculator was used to calculate the sample size with 99% confidence and a 1% margin of error [18]. The inclusion criteria for the 1,924 participants were adult citizens and non-Saudi residents over 18 years old, who had previously received two or three doses of the vaccine. Children and adolescents below 18 years old and those who had fewer than two doses of the vaccine constituted the exclusion criteria.

Questionnaire design

The questionnaire was distributed from October 1 to November 20, 2022. It was anonymous and justified to accept only a single response from the same person. The participants were informed that the survey responses guaranteed their anonymity. The participants did not receive any gifts or monetary compensation. The survey began with information about the title and objective of the study, followed by a statement informing participants that by completing the questionnaire, they were giving their approval to participate in the study. This was followed by a question about whether they agreed to participate in this study. If the answer was “yes,” they advanced to the next question. They were told that the form would not be submitted unless all questions had been answered. If the participant responded “no,” the questionnaire was ended. The survey included the following four sections: demographic data, disease knowledge, optimized questions of the standard Generalized Anxiety Disorder (GAD-7) assessment, and questions related to their acceptance of getting the vaccine if it were offered. The questionnaire was based on the standardized GAD-7 according to Spitzer et al. (2006) [19]. The survey was distributed among medical students and other social media including Twitter, Facebook, WhatsApp, and Snapchat.

The questions included the participants’ views on the following topics: (i) Feeling nervous, anxious, or on edge about getting the fourth dose; (ii) not being able to stop or control worrying about the short- and long-term side effects of the fourth dose; (iii) worrying too much about side effects and not being able to stop thinking about them; (iv) being so restless that it was hard to sit still when thinking about the fourth dose of the vaccine; (v) difficulty relaxing when thinking about the fourth dose of the vaccine; (vi) becoming easily annoyed or irritated, even for a short period, as a result of thinking about taking the fourth dose; and (vii) experiencing sudden panic in regard to taking the fourth dose to an incapacitating degree that made it impossible to perform daily activities. The anxiety score was calculated using an online calculation tool [20]. Briefly, if participants answered, “not at all,” they were then asked to rate the frequency based on 0, 1, 2, 3, for not at all, several days, more than half the days, and nearly every day, respectively. The sums were calculated, whereby 0-4 was considered normal, while scores of 5-9, 10-14, and >15 were considered mild, moderate, and severe, respectively.

Statistical analysis

Data were analyzed using the SPSS program software version 16 (SPSS Inc., Chicago, IL, USA). Frequencies and percentages were used for categorical data. Differences between groups were screened in crosstab analysis using the chi-square test. Furthermore, the question was asked, “If the fourth dose becomes available, would you want to take it?” Cronbach’s α was used to examine the internal consistency and reliability regarding GAD-7 questions.

## Results

The reliability of Cronbach’s α for the GAD-7 scale was found to be 0.878. A total of 1,924 participants completed the questionnaire and agreed to participate in the study. In contrast, only two participants declined the offer to participate in the study. Of the total respondents, 1,097 (57%) showed varying degrees of anxiety toward getting the fourth dose of the vaccine, while 827 (43%) showed no evidence of anxiety. Of the participants, 1,097 (57.0%) showed varying signs of anxiety, including mild anxiety, 531 (27.5%); moderate anxiety, 410 (21.35%); and severe anxiety, 156 (8.1%).

Significant differences were evident between genders and within age groups (Table 1). The present study indicated that females were significantly more affected than males, and those in the age range of 31-60 years were more affected than those aged 18 to 30 years. The age range of 31-40 years represented the largest group with expressed anxiety (67.6%), followed by those 41 to 50 years old with 63.4% (Table 1).

**Table 1:** The effect of age and sex on the anxiety level determined by (GAD-7). ^1^: The total number of respondents who exhibited mild-to-severe anxiety based on their GAD-7 scores. ^2^: Asymptotic significance two-sided. GAD-7 = Seven-Item Generalized Anxiety Disorder

Age (years)	Sex	GAD-7				Total affected ^1^	Cumulative	P-value^ 2^
		None (0–4)	Mild (5–9)	Moderate (10–14)	Severe (15 and more)			
18–20	Male	156 (51.2%)	91 (29.8%)	42 (13.8%)	16 (5.2%)	149 (48.9%)	305 (54.1%)	0.086
	Female	120 (46.3%)	69 (26.6%)	57 (22.0%)	13 (5.0%)	139 (53.7%)	259 (45.9%)	
	Total	276 (48.9%)	160 (28.4%)	99( 17.6%)	29 (5.1%)	288 (51.1%)	564 (100.0%)	
21–30	Male	171 (50.3%)	92 (27.2%)	55 (16.2%)	22 (6.5%)	169 (49.7%)	340 (47.2%)	0.008
	Female	144 (37.8%)	121 (31.8%)	85 (22.3%)	31 (8.1%)	237 (62.2%)	381 (52.8%)	
	Total	315 (43.7%)	213 (29.5%)	140 (19.4%)	53 (7.4%)	406 (56.3%)	721 (100%)	
31–40	Male	29 (36.2%)	29 (36.2%)	14 (17.5%)	8 (10.0%)	51 (63.8%)	80 (46.2%)	0.008
	Female	27 (29.0%)	20 (21.5%)	38 (40.9%)	8 (8.6%)	66 (71.0%)	93 (53.8%)	
	Total	56 (32.4%)	49 (28.3%)	52 (30.1%)	16 (9.2%)	117 (67.6%)	173 (100.0%)	
41–50	Male	51 (39.8%)	33 (25.8%)	30 (23.4%)	14 (10.9%)	77 (60.2%)	128 (55.4%)	0.009
	Female	22 (21.4%)	26 (25.2%)	41 (39.8%)	14 (13.6%)	81 (78.6%)	103 (44.6%)	
	Total	73 (31.6%)	59 (25.5%)	71 (30.7%)	28 (12.1%)	158 (63.4%)	231 (100.0%)	
51–60	Male	64 (48.9%)	28 (21.4%)	19 (14.5%)	20 (15.3%)	67 (51.1%)	131 (73.6%)	0.001
	Female	13 (27.7%)	10 (21.3%)	22 (46.8%)	2 (4.3%)	34 (72.3.8%)	47 (26.4%)	
	Total	77 (43.3%)	38 (21.3%)	41 (23.0%)	22 (12.4%)	101 (56.7%)	178 (100.0%)	
>61	Male	25 (51.0%)	12 (24.5%)	6 (12.2%)	6 (12.2%)	24 (49.0%)	49 (86.0%)	0.403
	Female	5 (62.5%)	0 (0.0%)	1 (12.5%)	2(25.0%)	3 (37.5%)	8 (14.0%)	
	Total	30 (52.6%)	12 (21.1%)	7 (12.3%)	8 (14.0%)	27 (47.4%)	57 (100.0%)	
Cumulative		827 (43.0%)	531 (27.6%)	410 (21.3%)	156 (8.1%)	1,097 (57%)	1,924 (100.0%)	

In this study, 448 (23.3%) participants received two doses and 1,476 (76.7%) completed three doses of the SARS-CoV-2 vaccine (Table 2). A total of 292 (65.2%) participants who received two doses showed signs of anxiety. The signs in this group were mild in 133 (29.7%) cases, moderate in 106 (23.7%), and severe in 53 (11.8%). Of those receiving three doses, 805 (54.5%) showed signs of anxiety, including 398 (27.0%) who were mild, 304 (20.6%) moderate, and 103 (7.0%) severe. There was a significant difference (p < 0.001) between the incidence of anxiety among different groups (Table 2).

**Table 2:** The effect of the type and number of the SARS-CoV-2 vaccine doses on the anxiety level determined by GAD-7. Chi-square: p < 0.001 (asymptotic significance two-sided). ^1^: The total number of respondents who exhibited mild-to-severe anxiety based on their GAD-7 scores. SARS-CoV-2 = severe acute respiratory syndrome coronavirus 2; GAD-7 = Seven-Item Generalized Anxiety Disorder

Number of doses	Types of vaccines	GAD-7				Total affected ^1^	Cumulative
		None (0–4)	Mild (5–9)	Moderate (10–14)	Severe (>15)		
Two doses	Two doses of Moderna	6 (50.0%)	1 (8.3%)	4 (33.3%)	1 (8.3%)	6 (50.0%)	12 (0.6%)
	Two doses of Pfizer	123 (34.3%)	106 (29.5%)	86 (24.0%)	44 (12.3%)	236 (65.7%)	359 (18.7%)
	Two doses of AstraZeneca	27 (35.1%)	26 (33.8%)	16 (20.8%)	8 (10.4%)	50 (64.9%)	77 (4.0%)
	Total	156 (34.8)	133 (29.7%)	106 (23.7%)	53 (11.8%)	292 (65.2%)	448 (23.3%)
Three doses	One dose of AstraZeneca/Two doses of Pfizer	139 (44.0%)	100 (31.6%)	52 (16.5%)	25 (7.9%)	177 (56.0%)	316 (16.4%)
	Three doses of Pfizer	441 (46.5%)	241 (25.4%)	201 (21.2%)	65 (6.9%)	507 (53.5%)	948 (49.3%)
	Two doses of AstraZeneca/One dose of Pfizer	59 (39.1%)	39 (25.8%)	43 (28.5%)	10 (6.6%)	92 (60.9%)	151 (7.8%)
	One dose of AstraZeneca/One dose of Pfizer/One dose of Moderna	32 (53.5%)	18 (29.5%)	8 (13.1%)	3 (4.9%)	29 (47.5%)	61 (3.2%)
	Total	671 (45.5%)	398 (27.0%)	304 (20.6%)	103 (7.0%)	805 (54.5%)	1,476 (76.7%)
Cumulative		827 (43.0%)	531 (27.6%)	410 (21.3%)	156 (8.1%)	1,097 (57.0%)	1,924 (100.0%)

There were significant variations in anxiety (p < 0.001) based on the level of education and health information. Healthcare workers showed the lowest level of anxiety relative to other groups; in fact, a high percentage of them (104, 58.4%) showed no degree of anxiety (Table 3).

**Table 3:** The effect of education level on the anxiety level determined by GAD-7. Chi-square: p < 0.001 (asymptotic significance two-sided). ^1^: The total number of respondents who exhibited mild-to-severe anxiety based on their GAD-7 scores. SARS-CoV-2 = severe acute respiratory syndrome coronavirus 2; GAD-7 = Seven-Item Generalized Anxiety Disorder

Variable	GAD-7				Total affected ^1^	Cumulative
	None (0–4)	Mild (5–9)	Moderate (10–14)	Severe (15 and more)		
Healthcare (student/worker)	104 (58.4%)	41 (23.0%)	24 (13.5%)	9 (5.1%)	74 (41.6%)	178 (9.3%)
Postgraduate studies	38 (40.0%)	19 (20.0%)	24 (25.3%)	14 (14.7%)	57 (60.0%)	95 (4.9%)
Bachelor	446 (41.6%)	306 (28.6%)	240 (22.4%)	79 (7.4%)	625 (58.4%)	1,071 (55.7%)
Secondary school	206 (42.0%)	135 (27.6%)	103 (21.0%)	46 (9.4%)	284 (58.0%)	490 (25.5%)
Preparatory school	26 (38.2%)	23 (33.8%)	13 (19.1%)	6 (8.8%)	42 (61.8%)	68 (3.5%)
Primary school	7 (31.8%)	7 (31.8%)	6 (27.3%)	2 (9.1%)	15 (68.2%)	22 (1.1%)
Cumulative	827 (43.0%)	531 (27.6%)	410 (21.3%)	156 (8.1%)	1,097 (57.0%)	1,924 (100.0%)

At the end of the questionnaire, we asked: “In case a fourth dose becomes available, would you agree to take it?” Surprisingly, only 555 (28.8%) of all participants (1,924) indicated that they would get the fourth dose, of whom 245 agreed, and 310 responded that they were not certain, but thought they would get it. A highly significant percentage of the participants (p < 0.001) did not want to get the fourth dose of the SARS-CoV-2 vaccine (Table 4). Interestingly, even those who showed no signs of anxiety based on the GAD-7 score did not want to get the fourth dose. They were either uncertain or felt they would probably not take the vaccine (271/827). Alternatively, some were certain that they would not take the vaccine (154/827). This constituted 32.8% and 18.6%, respectively, of the total number of participants who showed no signs of anxiety (Table 4).

**Table 4:** The response of people about their intention to get the fourth dose of the SARS-CoV-2 vaccine clustered based on their GAD-7 response. Chi-square: p < 0.001 (asymptotic significance two-sided). ^1^: The total number of respondents who exhibited mild-to-severe anxiety based on their GAD-7 scores. SARS-CoV-2 = severe acute respiratory syndrome coronavirus 2; GAD-7 = Seven-Item Generalized Anxiety Disorder

If the fourth dose is available, do you want to take it?	GAD7				Total affected ^1^	Cumulative
	None (0–4)	Mild (5–9)	Moderate (10–14)	Severe (15 and more)		
Yes	203 (82.9%)	29 (11.8%)	12 (4.9%)	1 (0.4%)	42 (17.1%)	245 (12.7%)
Not sure, but I think yes	199 (64.2%)	74 (23.9%)	35 (11.3%)	2 (0.6%)	111 (35.8%)	310 (16.1%)
Not sure, but I think no	271 (50.9%)	162 (30.0%)	85 (16.0%)	14 (2.6%)	261 (49.1%)	532 (27.7%)
No	154 (18.4%)	266 (31.8%)	278 (33.2%)	139 (16.6%)	683 (81.6%)	837 (43.5%)
Cumulative	827 (43.3%)	531 (27.6%)	410 (21.3%)	156 (8.1%)	1,097 (57.0%)	1,924 (100%)

**Figure 1:**
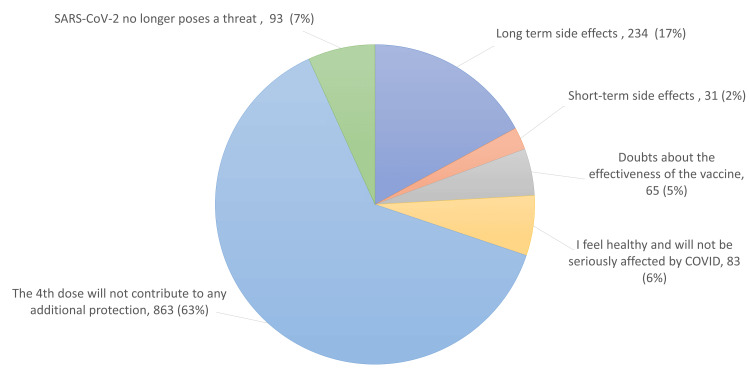
The frequency and percentage of potential causes of SARS-CoV-2 vaccine hesitancy. Individuals who exhibited reluctance in receiving the fourth dose of the SARS-CoV-2 vaccine tendered their justifications for abstaining from vaccination. SARS-CoV-2 = severe acute respiratory syndrome coronavirus 2

When we asked the participants who did not agree to get the vaccine (n = 1,369) about the cause of their hesitancy to get the vaccine, 234/ (17%) indicated that it was due to long-term side effects; 31 (2%) indicated it was due to the short-term side effects; 65 (5%) had doubts about the effectiveness of the vaccine; 83 (6%) felt sufficiently healthy to not be seriously affected by the SARS-CoV-2 infection; and 93 (7%) believed that SARS-CoV-2 no longer posed a real threat. However, the majority of respondents, 863 (63%), believed that the fourth dose would not contribute to any additional protection (Figure 1).

## Discussion

Many studies have discussed the development of anxiety and mental health-related diseases among different populations, including anxiety during the COVID-19 pandemic [21-23]. Anxiety has also been reported in regard to how long COVID-19 remains in the system after a person has been infected by SARS-CoV-2 [24].

Pre-existing anxiety and depression are strong progenitors of COVID-19-related mental health issues and reportedly increase stress, fear, and anxiety [23]. Both social and economic determinants could contribute to poor mental health [25]. This study revealed that anxiety from receiving the fourth dose of the SARS-CoV-2 vaccine is a significant concern, as such results were reported by more than half of the total participants.

The SARS-CoV-2 pandemic has resulted in millions of deaths worldwide. COVID-19 vaccines were successful in reducing fatalities among infected individuals. While vaccines are typically considered safe and effective, the rapid development and distribution of the SARS-CoV-2 vaccine has raised some concerns and increased anxiety and the hesitancy to receive vaccinations among some people.

We detected gender variations in anxiety levels regarding the fourth dose of the SARS-CoV-2 vaccine, and our results concur with previous reports that gender differences exist. Females generally reported significantly higher levels of anxiety related to SARS-CoV-2 infections than males [26]. Vaccine hesitancy was found to be more prevalent among women than men, although it did not specifically address anxiety related to the fourth dose of the SARS-CoV-2 vaccine [27,28].

Older people seem to have more confidence in SARS-CoV-2 vaccines. This finding was associated with the willingness of the general population to accept additional vaccine doses. In Southern Italy, a total of 438 participants, with ages ranging from 65 to 77 years, showed a high perception of the vaccine’s utility and had greater confidence in the information received about the second booster dose of the vaccine, although some respondents had lower trust in these sources of information. This discrepancy was explained by the lack of physicians’ recommendations, which was critical in helping people reach the correct decision [29].

In addition, adverse side effects from the SARS-CoV-2 vaccination and concerns about serious side effects after the vaccine were associated with vaccine hesitancy [30]. In this study, participants aged 31 to 40 years, followed by those aged 41-50 years, showed higher levels of anxiety than other age ranges. This finding agrees with another study that reported that older adults may be more prone to anxiety than younger adults [31]. However, other studies disagree with our findings and have reported that anxiety and vaccine acceptance are more likely to occur in younger adults than in older individuals [32-34]. Interestingly, in this study, people who received two doses of a SARS-CoV-2 vaccine presented more anxiety than those who received three doses. To our knowledge, there is currently no published evidence supporting this finding. It is possible that those who received a third dose of the vaccine may have experienced less anxiety because they did not experience severe side effects following the third vaccine. However, groups that received three doses of the SARS-CoV-2 vaccine still experienced considerable levels of anxiety.

More research is needed to fully understand the relationship between the number of vaccine doses and anxiety. In this study, 555 of the 1,924 participants agreed to get the fourth dose of the vaccine when it became available. The remaining participants did not want to get the fourth dose, including those who did not show any signs of anxiety based on their GAD-7 score. In previous studies, the percentage of individuals who were willing to take a third dose ranged from 44.6% to 95.5%, while the percentage of unwilling individuals ranged from 1.0% to 30.7% [35-38].

In another study in China, there was public acceptance of the COVID-19 vaccination. However, due to concerns about the safety of the vaccine, there was a significant decline in the intention to vaccinate immediately [39]. Vaccine hesitancy was also reported but with lower frequency among the Malaysian population, as more than 20% of the participants in the study showed hesitancy to get the second booster of the COVID-19 vaccine [40].

Study participants indicated reasons for their hesitancy, including long- and short-term side effects, doubts about the effectiveness of the vaccine, and the belief that they were sufficiently healthy and would not be seriously affected by the SARS-CoV-2 infection. Furthermore, participants believed that the SARS-CoV-2 no longer posed a real threat, and the majority of respondents believed that the fourth dose would not provide any additional protection.

However, vaccines were subjected to rigorous testing and clinical trials before approval. Additionally, regulatory agencies, such as the U.S. FDA and the European Medicines Agency, reviewed the results of these trials and other data to determine whether the vaccine was safe and effective for public use.

Another source of anxiety related to getting the SARS-CoV-2 vaccine was fear of short- and long-term side effects [24,41-44]. However, side effects only occur in rare situations, and the benefits of receiving the vaccine far outweigh the risks, as it can protect against death from severe COVID-19 illness [45]. The fear of side effects is a common concern for any vaccine, although the side effects are incomparable to the deleterious effects and fatal consequences of the SARS-CoV-2 infection.

It is worth noting that anxiety is not restricted or unique to the SARS-CoV-2 vaccine. Vaccine hesitancy and anxiety have been documented for flu vaccines, human papillomavirus vaccines, childhood vaccines, and many others [46-50]. However, the COVID-19 pandemic has brought these issues to the forefront and highlighted the need for effective communication and education to promote vaccine acceptance and reduce anxiety.

Many findings have shown that physicians’ directions to their patients are trustworthy and effective in fostering awareness about the importance of SARS-CoV-2 vaccines [29,51-53]. Accordingly, the evidence-based orientation of physicians in hospitals and primary healthcare about the importance of SARS-CoV-2 booster vaccination in saving lives and reducing the severity of the disease is crucial, as they constitute trusted sources of information.

Limitations

As in all studies, the current research has limitations. We used an electronic survey; therefore, people without access to the Internet could not participate. In addition, we did not incorporate the place of residence (rural or urban). In our investigation, we did not thoroughly evaluate the credibility or reliability of the information sources utilized by the participants in shaping their views on vaccination. This absence of source validation may introduce potential biases into our research. As GAD-7 has been well-established since 2006, we did not conduct a pilot study. Although this survey has limitations, to our knowledge, this study is the first to highlight the level of anxiety among people toward getting the fourth dose of the SARS-CoV-2 vaccine in Saudi Arabia and in the Middle East.

## Conclusions

The present study examined the GAD-7 scale in the context of individuals receiving their fourth dose of the SARS-CoV-2 vaccine. The study aimed to assess overall willingness to receive this vaccine. It was found that over half of the participants (57%) reported varying degrees of anxiety concerning the administration of the fourth dose. There was also an increased level of unwillingness and hesitancy to receive the SARS-CoV-2 vaccine. Social research is essential to mitigate anxiety among Saudi nationals and residents, enabling the implementation of effective strategies that provide reliable information about vaccine efficacy and safety. Additionally, addressing the social and economic factors that contribute to anxiety is crucial. Vaccine hesitancy and the spread of misinformation can have significant consequences, leading to reduced vaccination rates. Therefore, it is imperative to establish effective communication and raise awareness about the importance of receiving the fourth vaccine dose, particularly among priority groups such as the elderly, immunocompromised patients, and healthcare workers.
